# Neuronal Injury External to the Retina Rapidly Activates Retinal Glia, Followed by Elevation of Markers for Cell Cycle Re-Entry and Death in Retinal Ganglion Cells

**DOI:** 10.1371/journal.pone.0101349

**Published:** 2014-07-01

**Authors:** Alba Galan, Pauline Dergham, Pedro Escoll, Antonio de-la-Hera, Philippe M. D'Onofrio, Mark M. Magharious, Paulo D. Koeberle, José María Frade, H. Uri Saragovi

**Affiliations:** 1 Lady Davis Institute-Jewish General Hospital, Montreal, Quebec, Canada; 2 Department of Medicine, Molecular Medicine Institute (IMMPA CSIC/UAH), School of Medicine, Alcalá University, Alcalá de Henares, Madrid, Spain; 3 Graduate Department of Rehabilitation Sciences, University of Toronto, Toronto, ON, Canada; 4 Department of Surgery, University of Toronto, Toronto, ON, Canada; 5 Department of Molecular, Cellular and Developmental Neurobiology, Cajal Institute, CSIC, Madrid, Spain; 6 Department of Pharmacology and Therapeutics, McGill University, Montreal, Quebec, Canada; 7 Department of Oncology and the Cancer Center, McGill University, Montreal, Quebec, Canada; Universidade Federal do Rio de Janeiro, Brazil

## Abstract

Retinal ganglion cells (RGCs) are neurons that relay visual signals from the retina to the brain. The RGC cell bodies reside in the retina and their fibers form the optic nerve. Full transection (axotomy) of the optic nerve is an extra-retinal injury model of RGC degeneration. Optic nerve transection permits time-kinetic studies of neurodegenerative mechanisms in neurons and resident glia of the retina, the early events of which are reported here. One day after injury, and before atrophy of RGC cell bodies was apparent, glia had increased levels of phospho-Akt, phospho-S6, and phospho-ERK1/2; however, these signals were not detected in injured RGCs. Three days after injury there were increased levels of phospho-Rb and cyclin A proteins detected in RGCs, whereas these signals were not detected in glia. DNA hyperploidy was also detected in RGCs, indicative of cell cycle re-entry by these post-mitotic neurons. These events culminated in RGC death, which is delayed by pharmacological inhibition of the MAPK/ERK pathway. Our data show that a remote injury to RGC axons rapidly conveys a signal that activates retinal glia, followed by RGC cell cycle re-entry, DNA hyperploidy, and neuronal death that is delayed by preventing glial MAPK/ERK activation. These results demonstrate that complex and variable neuro-glia interactions regulate healthy and injured states in the adult mammalian retina.

## Introduction

Recent reports have shown that, following injury, post-mitotic neurons can reactivate the cell cycle and enter the S-phase to produce DNA hyperploidy and hypertrophy. In post-mitotic neurons, cell cycle proteins are normally down-regulated and re-entry into the cell cycle presumably leads those cells into apoptosis. In contrast, cells such as astrocytes and glial cells retain mitotic potential and the re-expression of cell cycle genes leads to successful cell cycle re-entry and proliferation [1], [2].

Here, we use a model of full transection or axotomy of the optic nerve (ON) to study the reciprocal cross-talk between the injured neurons and the uninjured retinal glia. The ON is composed of fibers projecting to the brain from neuronal retinal ganglion cells (RGCs) whose cell bodies are in the retina. Thus, the ON injury is extra-retinal, in a different anatomical compartment from where the RGC somata are located. In addition, the retina is a highly ordered, multilayered system with the RGC soma residing in the inner layers, the photoreceptors in the outer layers, and additional neurons intermingled with glia and Müller cells in the intervening space [3].

While ON axotomy only transects RGC axons, it has effects on the other cellular compartments of the retina. Thus ON axotomy is a useful model to study neurodegeneration in different anatomical and cellular compartments of the retina after extra-retinal injury to RGC fibers [4]. Following ON axotomy, the injury signals travel retrogradely to the RGC somata located in the retina, eventually causing RGC death over time [5]–[7]. Here we report on intracellular signals in glia and neurons, that precede RGC death, and the associated molecular events that lead to neuronal cell cycle re-entry, DNA hyperploidy, and RGC death after ON axotomy.

## Materials and Methods

### Animals and anesthesia

All animal procedures respected the IACUC guidelines for use of animals in research, and to protocols approved by McGill University Animal Welfare Committees. Wistar female rats (250–300 g, Charles River) were housed 12 hour dark-light cycle with food and water *ad libitum*. Deep anesthesia (ketamine, xylazine, acepromazine injected intraperitoneally; 50/5/1 mg/kg, as per IACUC recommendations) was used for ON axotomy, fluorogold retrograde labeling, and euthanasia.

### Animal model

#### Optic nerve axotomy

The procedure was done as described [5], [8], with one modification. We performed the ON transection at 2.0 mm instead of 1.0 mm as we have reported previously [7]. This provided us a better experimental time window for our studies, because longer distances from injury caused delayed RGC cell body death (seven days after axotomy ∼35% RGC death at 2.0 mm *versus* 50% RGC death at 1.0 mm). Briefly, a 1.5–2.0 cm skin incision was made along the edge of the right orbit bone; lachrymal glands, orbital fats were excised and extraocular muscles were separated to expose the ON. An 18G needle was used to lacerate the sheath longitudinally in order not to disturb the ophthalmic artery; the ON parenchyma was then separated out and lifted by a homemade hook, and then completely transected 2.0 mm posterior to eyeball with micro-tweezers.

### Drug treatment in vivo

Intravitreal injections of the MAPK/ERK inhibitor PD98059 or control vehicle were as described [9], 1 hour after axotomy. Animals were placed in a stereotaxic frame and anesthetized with isoflurane, delivered through a gas anesthetic mask. The cornea was anesthetized using Alcaine eye drops (Alcon) before intraocular injections. A pulled glass micropipette attached to a 10 µl Hamilton syringe via a hydraulic coupling through PEEK tubing was used to deliver 4 µl of a solution into the vitreous chamber of the eye, posterior to the limbus. Care was taken to prevent damage to the lens or anterior structures of the eye that have been shown to secrete confounding growth factors. The pipette was held in place for 5 s after injection and slowly withdrawn from the eye to prevent reflux. Injections were performed using a surgical microscope to visualize pipette entry into the vitreous chamber and confirm delivery of the injected solution.

### Fluorogold (FG) Retrograde Labeling

RGCs were retrogradely labeled *in vivo* with a 4% FG solution (Flurochrome, Englewood, CO) applied bilaterally to the superior colliculous (SC) as previously described [10]. Briefly, rats were mounted on stereotactic apparatus (Kopf Instruments, Tujunga, CA), holes were drilled at a position 1.3 mm lateral to the sagital suture and 2.5 mm anterior to lambda suture on each side, and FG (3 µl) was injected into the SC at the depth of 6.0 mm bellow the skull. Then, holes were then filled with gelfoam soaked in 4% FG. This technique was used to specifically visualize RGCs for analyses of cell cycle proteins, and for quantification of RGC death.

### Immunohistochemistry

After enucleation, the eyes were immersed overnight in fixative composed of 4% paraformaldehyde [11] in PBS (pH 7.4) at 4°C, followed by cryoprotection by soaking in 30% sucrose overnight at 4°C. Eyes were frozen in dry ice, and cryostat sections were cut and mounted onto gelatin-coated glass slides. Sections (12 or 20 µm thick) were washed with phosphate-buffered saline (pH 7.4, PBS) and then, incubated in PBS containing 10% normal goat serum, 0.3% Triton X-100 and 0.5% bovine serum albumin (BSA) for 2 hours. After, sections were incubated overnight at 4°C either with primary antibody (Table 1). The sections were rinsed and incubated with secondary antibody (Table 1) for 1–2 hours at room temperature. After washing, the sections were cover-slipped using Immuno-Mount (Shandon, PA, USA) or glycerol/PBS (1∶1).

**Table 1:** Details of antibodies used for immunohistochemistry.

Primary antibodies				
Specificity	Source	Clone	Company	Dilution
Anti-glial fibrillary acid protein (GFAP)	Rabbit	Polyclonal	Millipore	1∶300
CRALBP	Mouse	B2	Cedarlane	1∶200
Cyclin A	Mouse	CY-A1	Sigma	1∶500
Cyclin D3	Mouse	Monoclonal	Cell Signaling	1∶200
E2F1	Rabbit	Polyclonal	Santa Cruz	1∶200
NeuN	Mouse	A60	Millipore	1∶40
p44/p42 MAPK (Erk1/2)	Rabbit	Polyclonal	Cell Signaling	1∶25
Phospho p44/p42 MAPK (Erk1/2) (Thr202/Tyr204)	Rabbit	Polyclonal	Cell Signaling	1∶200
Phospho-Akt (Ser473)	Rabbit	D9E	Cell Signaling	1∶100
Phospho-retinoblastoma (pRb)	Rabbit	Polyclonal	Cell Signaling	1∶200
Phospho-S6 (pS6)	Rabbit	Polyclonal	Cell Signaling	1∶1000
Retinoblastoma	Mouse	Monoclonal	BD Pharmigen	1∶400
Retinoblastoma	Mouse	4H1	Cell Signaling	1∶200

Secondary antibodies				
Specificity	Source	Conjugate	Company	Dilution
Anti-Rabbit IgG	Goat	Alexa Fluor 488	Invitrogen	1∶200
Anti-Mouse IgG	Goat	Alexa Fluor 594	Invitrogen	1∶200
Anti-Mouse IgG	Donkey	Rhodamine Red-X	Jackson ImmunoResearch	1∶200

### Image acquisition

Pictures were taken as Z-stacks of confocal optical sections using a Leica confocal microscope equipped with argon and helium neon lasers applying a 20X objective. Images were exported directly in TIF format and adjusted using Adobe Photoshop CS 8.0 for unbiased brightness and contrast.

### Immunohistochemistry data analysis

The total number of NeuN or FG-labeled cells were quantified for each axotomized (OD) *versus* contralateral uninjured (OS) eyes at the time points indicated. Then, the number of NeuN^+^–p-S6^+^ cells, FG^+^–pRb^+^ cells and FG^+^–cyclin A^+^ cells were counted manually in the RGC layer. For each experimental condition, a minimum of 10 images were acquired from at least 3 sections cut from different areas of the retina (n = 7 retinas per group). Data were shown as percentage of FG^+^ or NeuN^+^ cells for each of the corresponding primary antibodies (p-S6, p-Rb or cyclin A) ±SEM.

The area of the profiles of the cells expressing p-Akt and the p-ERK intensity immunoreactivity were measured using ImageJ software (developed by Wayne Rasband, National Institutes of Health, Bethesda, MD, USA; http://imagej.nih.gov/ij). Data are shown as the average area (in pixels) in unijured (OS) *versus* axotomized (OD) eyes at three days after axotomy for p-Akt (n = 9 retinas per group) or as the average intensity (in pixels) in vehicle *versus* PD98059 three and five days after axotomy for p-ERK (n = 4 retinas per group).

### Analysis of DNA content (Slide-based cytometry; SBC)

FG-labeled retinas were dissected, fixed with 4% PFA, and incubated twice with PBS/0.1% Triton X-100 for 30 min each. DNA was then stained for 1 min with 50 µg/ml propidium iodide (PI; Sigma) in PBS. Then, retinas were washed five times with PBS, and flat-mounted in glycerol/PBS (1∶1). The relative DNA content of the FG-labeled RGCs was determined as a measure of the integral PI fluorescence values (i.e. integration of total pixel intensity assigned to the visualized nuclei) following a method previously described [12]. This analysis was performed using the automated Olympus IX81 microscope-based imaging platform (Olympus), equipped with a digital camera (ORCA-AG C8484-05G01, Hamamatsu Photonics), using 20x magnification objectives. A minimum of 25 fields were acquired in each retina. Analysis of acquired images was performed using Scan^R^ Analysis software (Olympus). Intensity modules were used for setting the main object “cell soma” (FG staining) and the secondary object “cell nucleus” (PI staining). Basically, cytometry-orientated data analysis was used, setting a first gate of single cells, excluding small particles (less than 30 µm^2^) or doublets with a dot-plot of area versus circularity. Then, a histogram of PI intensity from the gate of single cells was obtained.

### Analysis of DNA content and RGCs size (Fluorescence microscopy)

The DNA content of FG-labeled RGCs in flat-mounted retinas was determined by measuring the PI intensity with “Metamorph” software (Molecular Devices). For each retina (n = 3), overlayed images of FG and PI-labeling were created, and PI intensity per pixel ±SEM was measured in FG^+^ RGCs. FG^+^ PI^+^ RGCs were classified as “small” and “large” depending on their diameter in pixels. Cells with values between 100 and 400 pixels were considered “small” and cells with values between 400 and 1000 were consider “large”. More than 7000 cells per group were counted and all retinal areas (see next section) were averaged for each group. Debris and dead cells were excluded by setting up the threshold levels under 100 pixels for each image.

### Quantification of RGCs

Quantification of FG-labeled RGCs was performed as reported previously [5], [8], [13]. At the experimental end point, both eyes were enucleated; the anterior parts were cut out and the remaining part was fixed in 4% PFA for 30 minutes, were flat-mounted on a glass slide and dissected in four radial cuts to facilitate flattening of the retinas into a Maltese cross shape, with the vitreous side up. Pictures for each retina were taken using a fluorescence microscope (Carl Zeiss Meditec, Jena, Germany), with 12 pictures/retina at ×20 magnification. For each quadrant there were 3 pictures at a radial distance of 1, 2, and 3 mm from the central area (where the optic nerve exits the retina). These areas correspond to central, middle and peripheral retinal areas, respectively. In this model, RGC death is progressive. At day 1 post-axotomy there is no detectable death, at day 3 post-axotomy there is 6±3% RGC death, at day 5 post-axotomy there is 15±4% RGC death (p<0.05), and at day 7 post-axotomy there is 36±5% RGC death (p<0.01). Automated RGC quantitative counting was done with “Metamorph” software (Molecular Devices) as reported before [7].

### Statistical analysis

Was conducted using Student t-test. After standardizing experimental (right eye) and the contralateral control eye in a single animal, and averaging all animals for a group ±SEM.

## Results

### ON axotomy causes progressive death of RGCs

ON axotomy is an extra-retinal injury that affects the neuronal RGC population, whose axons form the optic nerve. When the ON is completely transected RGC death is progressive. In the paradigm used here, at day 1 post-axotomy there is no detectable RGC death, at day 3 post-axotomy there is 6±3% RGC death, at day 5 post-axotomy there is 15±4% RGC death (p<0.05), and at day 7 post-axotomy there is 36±5% RGC death (p<0.01). Similar data have been published by us and other laboratories.

In this paradigm, we present data on the time kinetics of the changes in signaling, cell cycle, and cell biological events that take place in retina (and specifically in RGCs and Müller cells) after ON axotomy.

### Axonal injury rapidly decreases p-S6 in RGCs

**Figure 1:**
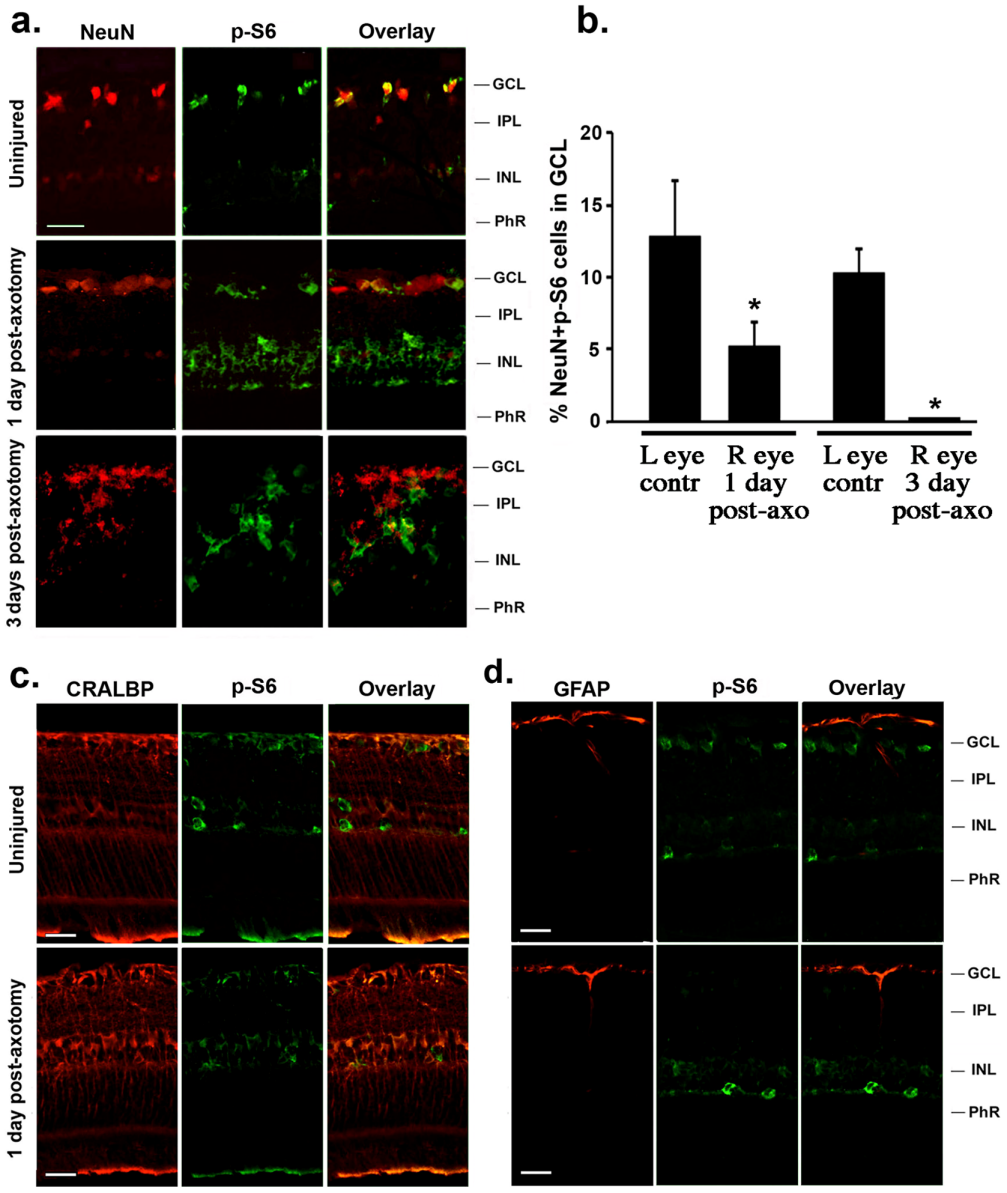
Expression of phospho-S6 (p-S6) decreases in the GCL after axotomy. (**a**) p-S6 immunoreativity in neurons of the GCL. In control uninjured retinas, p-S6 immunoreactivity was detected in some neurons in the GCL (likely RGCs) and other cell types in the INL (likely horizontal cells and microglia). One day after axotomy, p-S6 decreased in the GCL, and three days after axotomy it was undetectable in neurons of the GCL. p-S6 did not change in neurons of the INL. (**b**) Quantification of NeuN^+^ p-S6^+^ cells in the GCL, comparing uninjured control (left) to injured (right) eye in each animal at 1 and 3 days after axotomy, total of >40 images taken from n = 4 retinas per group, *p<0.01. (**c, d**) lack of p-S6 immunoreactivity in glia. Neither astrocytes (labeled with GFAP) nor Müller cells (labeled with CRALBP) were p-S6^+^ 1 day after axotomy. GCL: ganglion cell layer; IPL: inner plexiform layer; INL: inner nuclear layer; PhR: photoreceptor layer. Scale bar, 30 µm.

p-S6 regulates mTOR-dependent pathways [14] and impacts global translation of mRNAs [15]. In uninjured control retinas p-S6 immunoreactivity was detected in NeuN^+^ RGCs. At post-axotomy day 1 RGCs had 65% decreased p-S6 (p<0.01) and at day 3 the p-S6 label was undetectable in RGCs (Figure 1a and 1b). Similar data were obtained by specifically labeling RGCs with FG rather than NeuN (data not shown).

Neither astrocytes nor Müller cells expressed p-S6 early after axotomy, as shown by the lack of co-localization of p-S6 with GFAP (astrocyte marker) or CRALBP (Müller cell marker) (Figure 1d and 1c).

However, other p-S6^+^ cells were detected in the INL. They were not identified (as they are not germane to this study) but likely they are microglia and horizontal cells based on their location and morphology. After injury in these cells the p-S6 immunoreactivity either increased slightly (Figure 1a, putative microglia) or remained unchanged (Figure 1a, 1c and 1d; putative horizontal cells).

### Axonal injury rapidly activates p-Akt specifically in retinal glia

**Figure 2:**
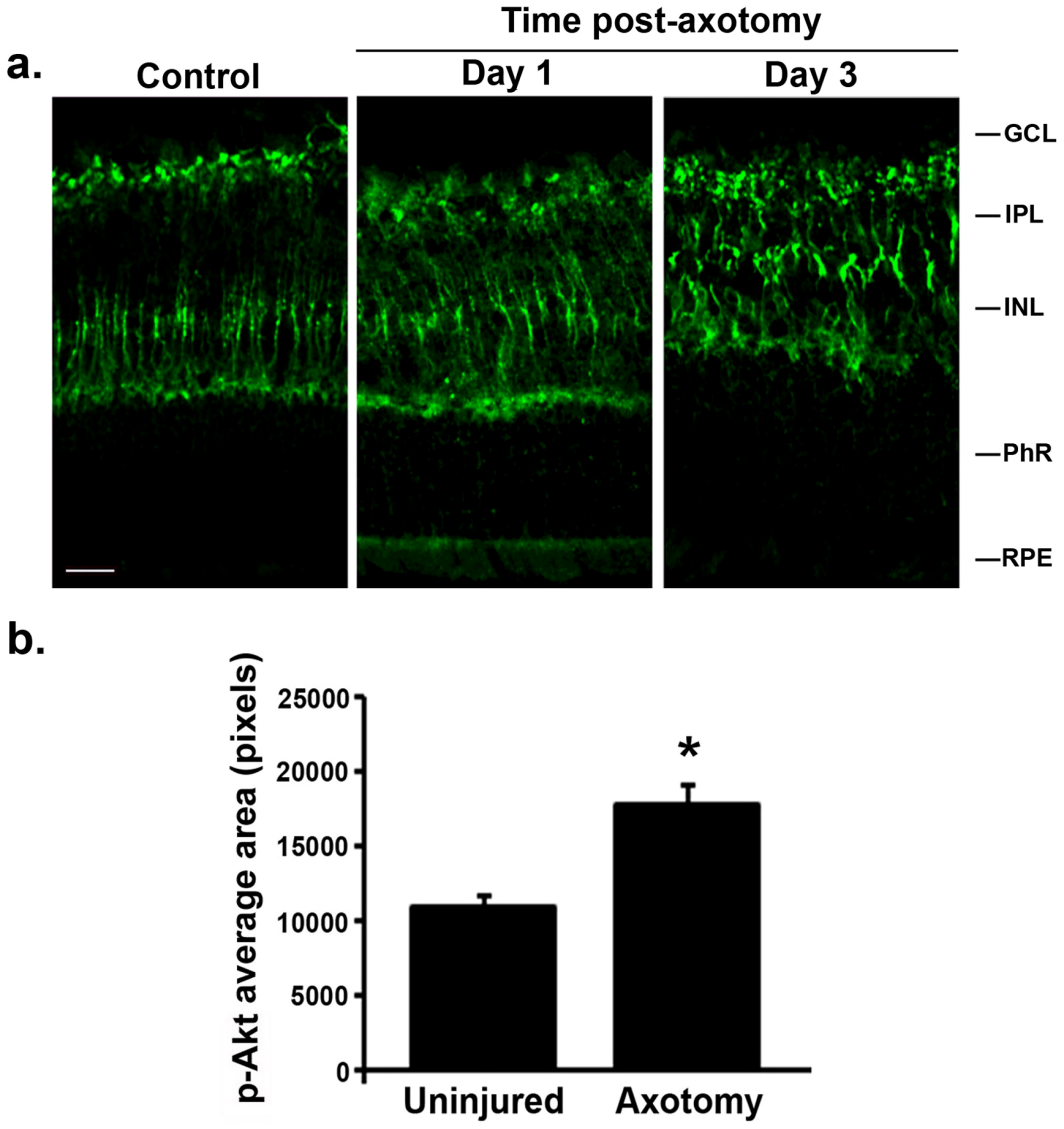
Up-regulation of phospho-Akt (p-Akt) after axotomy has a vectorial orientation towards the RGCs. (**a**) p-Akt immunoreactivity in control uninjured retinas, and at day 1 and day 3 after axotomy. No other antibody was used, to depict clearly the specific p-Akt reactivity. (**b**) Quantification of p-Akt area in uninjured and day 3 post-axotomy retinas. Area was significantly increased in axotomized eyes, as a sign of gliosis and hypertrophy of putative Müller cells after injury, >90 images taken from n = 9 retinas per group, *p<0.01. GCL: ganglion cell layer; IPL: inner plexiform layer; INL: inner nuclear layer; PhR: photoreceptor layer. Scale bar, 30 µm.

pAkt is also a surrogate marker of mTORC1 activity [16], and regulates cell growth and survival [17]. In uninjured control retinas p-Akt immunoreactivity was detected in putative Müller cells (Figure 2a). The p-Akt pattern showed smooth and long processes ending in strongly labeled basal end-feet, which lie adjacent to and surrounding the RGCs.

**Figure 3:**
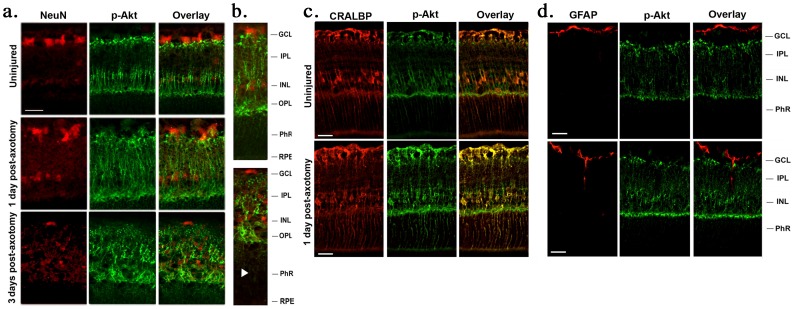
After axotomy phospho-Akt is increased in Müller cells. (**a–c**) In control and in experimental retinas p-Akt immunostaining was exclusively detected in Müller cells, based on their typical morphology and co-localization with CRALBP. One day post-axotomy, p-Akt staining increased in the processes of Müller cells. Three days post-axotomy, Müller cells showed signs of gliosis and strong p-Akt immunoreactivity. (**b**) Micrographs of the full thickness of the retinas, showing p-Akt immunostaining in Müller cell soma, and exclusively in the Müller cell fibers oriented towards the injured RGCs (top section, GCL). Arrowheads indicate that Müller cell fibers with p-Akt immunostaining are not oriented towards the photoreceptors (bottom section, PhR and RPE). (**d**) p-Akt is not expressed in astrocytes. P-Akt immunoreactivity was not detected in GFAP^+^ cells in both control and experimental retinas. GCL: ganglion cell layer; IPL: inner plexiform layer; INL: inner nuclear layer; OPL: outer plexiform layer; PhR: photoreceptor layer; RPE: retinal pigmented epithelium. Scale bar, 30 µm.

The identity of putative Muller cells was confirmed by co-staining with CRALBP (Figure 3d). At day 1 post-axotomy, Müller cell bodies and processes were more robustly p-Akt^+^ (Figure 2a, Figure 3a and 3c). At day 3 post-axotomy there was a noticeable hypertrophy of Müller cells, with a 40% increased area of p-Akt label (p<0.01 versus uninjured retinas) (Figure 2b and Figure 3a, 3b and 3c). p-Akt immunoreactivity was not detected in astrocytes or neurons, as revealed by the lack of co-localization with GFAP and NeuN (Figure 3a and 3d).

The data indicate that after ON axotomy p-Akt is specifically up-regulated exclusively in Müller cells. Curiously, p-Akt was localized at the Müller cell soma as well as their inner fibers and processes projecting towards the GCL, where the injured RGC cell bodies reside (Figure 3a, 3b, and 3c). However, the fibers that Müller cells project towards photoreceptors were p-Akt negative (Figure 3b). This suggests the existence of a specific distribution or maintenance of a polarized p-Akt signal which is oriented towards the injured RGC neurons but not towards the photoreceptor neurons. Such a polarized pattern of p-Akt staining in Müller cells could be caused by a gradient arising from injured RGCs.

### Axonal injury rapidly activates p-ERK1/2 specifically in retinal glia

**Figure 4:**
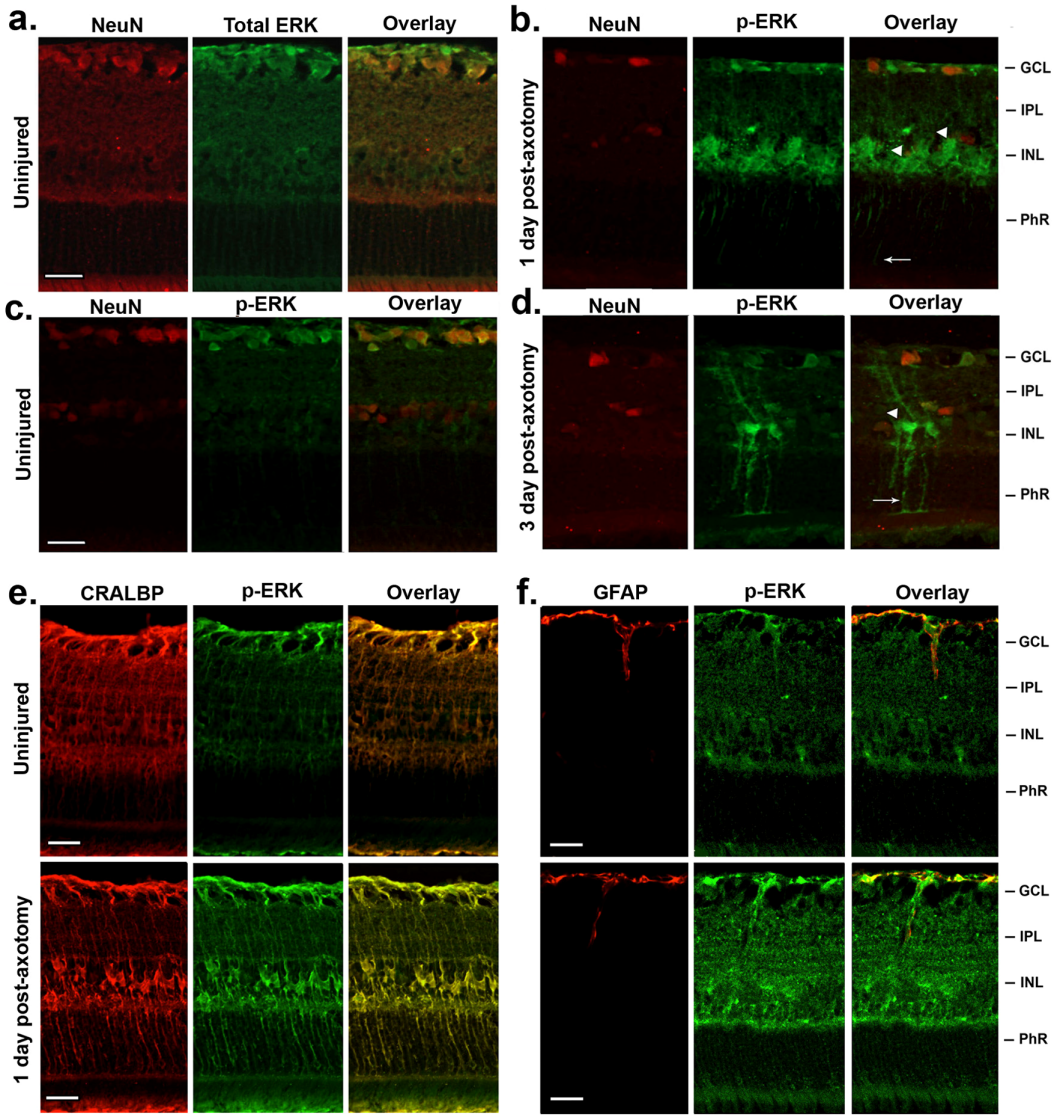
Retinal glia rapidly phosphorylate ERK1/2 after RGC axotomy. (**a–d**) p-ERK immunoreactivity in neurons (**a**) In uninjured retinas there is expression of ERK1/2 in all cellular compartments, both in neuronal and in non-neuronal cells, detected with an antibody to total ERK1/2. Total ERK1/2 staining continued to be expressed to the same level in retinas at days 1 and 3 post-axotomy (data not shown). (**c**) In uninjured retinas ERK1/2 is phosphorylated in RGCs. (**b**) At day 1 after ON axotomy, there was a robust increase of p-ERK1/2 immunoreactivity in putative glial cells in the GCL, INL, and both plexiform layers. Note the relative reduction of p-ERK1/2 in the GCL versus control normal retinas. (**d**) At day 3 after ON axotomy both the soma and the processes of retinal glia are robustly labeled with p-ERK1/2. Note that, unlike what was shown for p-Akt, the glial fibers with p-ERK1/2 point both towards the injured RGCs and towards the photoreceptors (full arrows). Arrowheads point to the soma of glia. (**e,f**) p-ERK immunoreactivity in glia. (**e**) p-ERK is strongly expressed in the end-feet, somas and processes of Müller cells expanding throughout the PhR layer rapidly after axotomy, as shown by co-localization of p-ERK with CRALBP. (**f**) The expression of p-ERK was low in astrocytes, as revealed by the weak co-localization of p-ERK with GFAP. GCL: ganglion cell layer; IPL: inner plexiform layer; INL: inner nuclear layer; PhR: photoreceptor layer. Scale bar, 30 µm.

Next, we studied p-ERK1/2 because its signals are associated with p-S6 signals (which are decreased in axotomized RGCs) and with p-Akt signals (which are increased in Müller cells, and polarized towards RGCs). In uninjured retinas total ERK1/2 protein is present in all cellular compartments, both in neuronal and in non-neuronal cells (Figure 4a). After ON axotomy there were no detectable changes in total ERK1/2 expression or distribution (data not shown). However after ON axotomy changes are seen in the phosphorylated form of ERK1/2 (p-ERK1/2).

In uninjured retinas p-ERK1/2 was detectable, including in RGCs and other cell types (Figure 4c). At day 1 post-axotomy there was a reduction of p-ERK1/2 in NeuN^+^ neurons (Figure 4b), but there was a robust increase of p-ERK1/2 in Müller cells (arrowheads) (Figure 4b and 4e). At day 3 post-axotomy the levels of p-ERK1/2 were markedly increased in the somata and processes of Müller cells (Figure 4d). p-ERK1/2 was not detected in astrocytes (Figure 4f).

The p-ERK1/2 positive Müller cell fibers were oriented towards the injured RGCs as well as the photoreceptors (Figure 4d, arrows). Hence, the localization of p-ERK1/2 in Müller cells is not polarized as had been observed for p-Akt.

### Axonal injury causes phosphorylation of Rb in post-mitotic neurons, but not in glia

p-S6, p-Akt and p-ERK1/2 immunoreactivity increased in glial cells after ON injury. Given that these proteins regulate cell division and cell growth, and that injury is known to induce gliosis, we studied whether axotomy affects the cell cycle. We studied expression of the cell cycle regulators retinoblastoma (total Rb versus phosphorylated-Rb, cell cycle re-entry), E2F1 (a transcription factor bound to Rb, released when Rb is phosphorylated), and cyclin D3 and cyclin A (cell cycle re-entry). These studies were done with retinas from uninjured control eyes *versus* post-axotomy days 1, 3 and 5.

**Figure 5:**
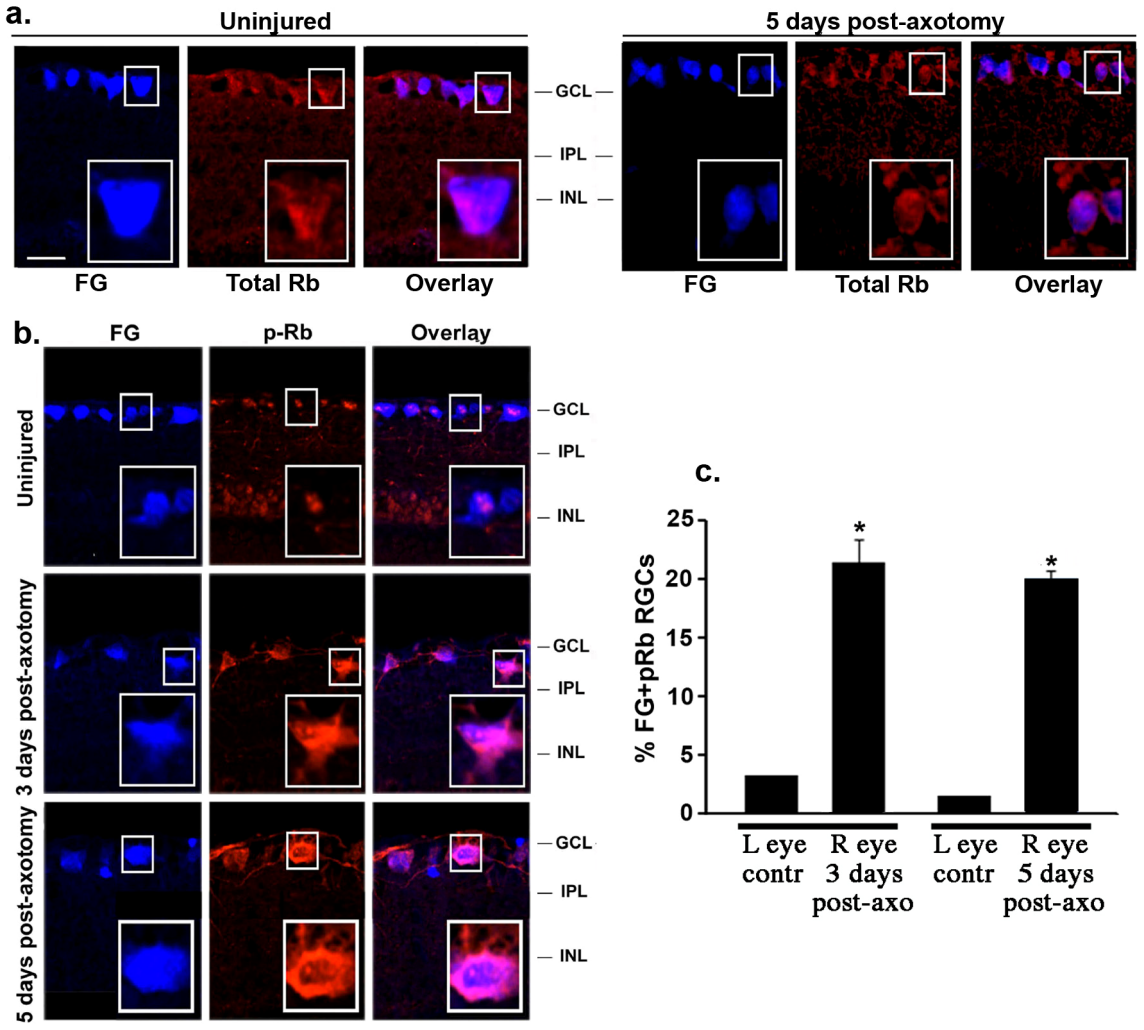
RGCs rapidly phosphorylate Rb after axotomy: marker of G1 cell cycle re-entry. (**a**) Total Rb expression was found in adult RGCs labeled with fluorogold (FG). No differences were observed between uninjured control and axotomized eyes, and moreover after axotomy there is no substantial change in total Rb in RGCs. In each picture, an RGC indicated with a small white rectangle is shown enlarged (lower right corner). (**b**) FG (RGC marker) and p-Rb immunostaining. In control uninjured retinas, a few RGCs showed weak p-Rb staining. At 3 and 5 days post-axotomy, p-Rb increased dramatically in RGCs (with no change in total Rb, see panel a). In each picture, an enlarged photomicrograph of an RGC indicated with a small white rectangle is shown. (**c**) Quantification of FG^+^ p-Rb^+^ RGCs in uninjured control (left) to injured (right) eye in each animal at 3 and 5 days after axotomy, total of >70 images taken from n = 7 retinas per group, *p<0.001. Scale bar, 30 µm. GCL: ganglion cell layer; IPL: inner plexiform layer.; INL: inner nuclear layer. FG: fluorogold.

The presence of total Rb in RGCs was specifically analyzed in neurons labeled retrogradely with FG (i.e. only RGCs are labeled). Total Rb showed a uniform, diffuse staining pattern in the cell bodies of RGCs, in both normal and injured eyes (Figure 5a). Identical results were obtained using a different antibody to total Rb, and by immunolabeling neurons with NeuN (data not shown). Together, these data indicate that after axotomy there are no changes in total Rb in RGCs.

Next, we evaluated the presence of p-Rb specifically in RGCs labeled with FG (Figure 5b). At post-axotomy day 1 p-Rb immunoreactivity was similar to uninjured control retinas, with few labeled RGCs (data not shown). At post-axotomy days 3 and 5, there was a robust increase of p-Rb labeling in RGCs, detected mainly in the cytoplasm and in some axons (Figure 5b). Quantification showed an increase of ∼20% of p-Rb^+^ RGCs after ON axotomy, whereas only ∼4% of total RGCs were p-Rb^+^ in uninjured control retinas (p<0.01) (Figure 5c).

The data revealed two surprising results. First, there were no detectable increases in p-Rb immunoreactivity in glia or in Müller cells up to day 5 after axonal injury, suggesting that they may not have initiated cell division. This may mean that increased p-S6, p-Akt, and p-ERK1/2 in glia and Müller cells may lead to cell growth (increase in cell size or hypertrophy). Second, increased Rb phosphorylation takes place in neurons (with no up-regulation of total Rb), suggesting that injured post-mitotic neurons may have initiated cell division.

### Cell cycle re-entry in post-mitotic neurons after axonal injury

Phosphorylation of Rb is a surrogate marker of G1 cell cycle re-entry. Phosphorylation of Rb results in the dissociation of the Rb/E2F complex and the activation of the E2F transcription factor [2], [18]. Thus, we examined whether E2F is expressed in RGCs.

**Figure 6:**
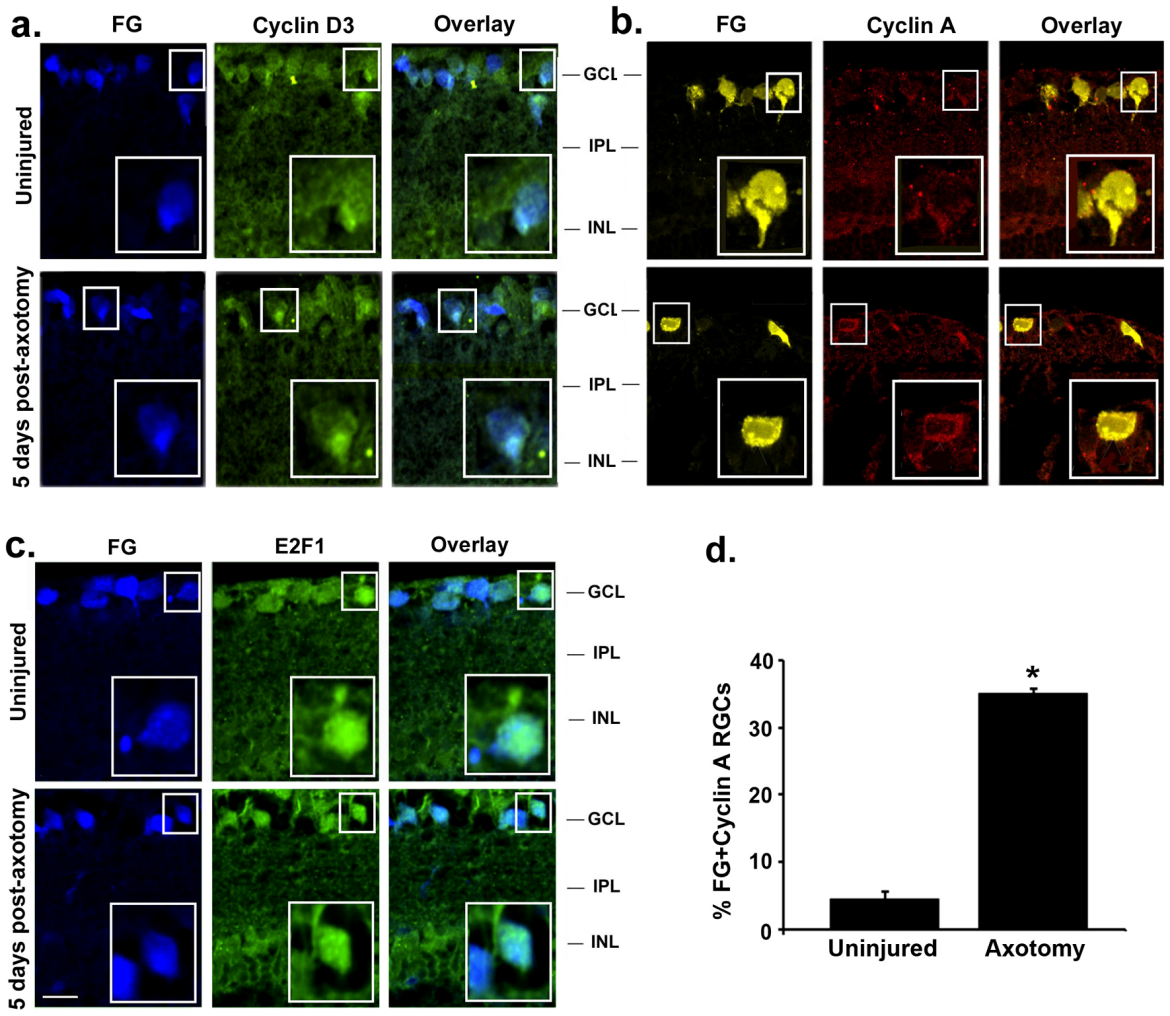
RGCs re-express Cyclin A after axotomy: marker of S-phase cell cycle re-entry. (**a**) Cyclin D3 immunostaining in FG-labeled RGCs. Control and injured retinas had similar staining. (**c**) E2F1 transcription factor immunostaining in FG-labeled RGCs. E2F1 was found in RGCs. Control and injured retinas had similar staining. (**b**) Cyclin A immunostaining in FG–labeled RGCs. Low expression of cyclin A was found in RGCs of control uninjured eyes. At day 5 post-axotomy, stronger cyclin A immunoreactivity was detected in some RGCs. For each picture, an RGC indicated with a small white rectangle is shown enlarged (lower right corner). (**d**) Quantification of FG^+^ Cyclin A^+^ RGCs in uninjured and axotomized eyes, 5 days after axotomy, total of >70 images taken from n = 7 retinas per group, *p<0.001. FG: fluorogold, GCL: ganglion cell layer; IPL: inner plexiform layer; INL: inner nuclear layer. Scale bar 30 µm.

E2F1 immunoreactivity was found in FG-labeled RGCs (Figure 6c). We note that control uninjured and injured eyes had the same intensity of E2F1 staining, in RGCs and in other cell types. This is likely because the anti-E2F1 antibody does not discriminate between “free” and “active” E2F1 and the E2F1 within Rb/E2F1 complexes. Nonetheless, Rb phosphorylation after injury is documented to cause the release of E2F1.

Cyclin A is a marker for the S-phase of the cell cycle [19]. In uninjured control eyes cyclin A immunoreactivity was undetectable in RGCs. However, after axotomy cyclin A immunoreactivity was increased in the RGC somata (∼35±0.74% of the FG-labeled RGCs were cyclin A^+^ after axotomy compared to uninjured eyes, p<0.001) (Figure 6b and Figure 6d), suggesting that axotomy may initiate S-phase re-entry in RGCs.

Together, these data indicate that components are present in injured RGCs to re-enter the cell cycle (p-Rb, E2F1, and Cyclin A). Cell cycle re-entry is a surprising event for adult post-mitotic neurons that are not supposed to replicate; but has been reported [20].

As additional controls, we also studied expression of D-type cyclins in RGCs. D-type cyclins activate cyclin-dependent-kinases (CDKs) that can phosphorylate Rb [18], [21]. We had reagents to study cyclin D3. However, there were no detectable differences in cyclin D3 immunostaining between uninjured control and axotomized eyes at any of the time points studied (Figure 6a, and day 3 post-axotomy data not shown). This suggests that the levels of cyclin D3 were not affected by axotomy, and that the generation of p-Rb in injured post-mitotic RGCs is independent of cyclin D3.

### Injured post-mitotic neurons that re-entered the cell cycle increase DNA content

**Figure 7:**
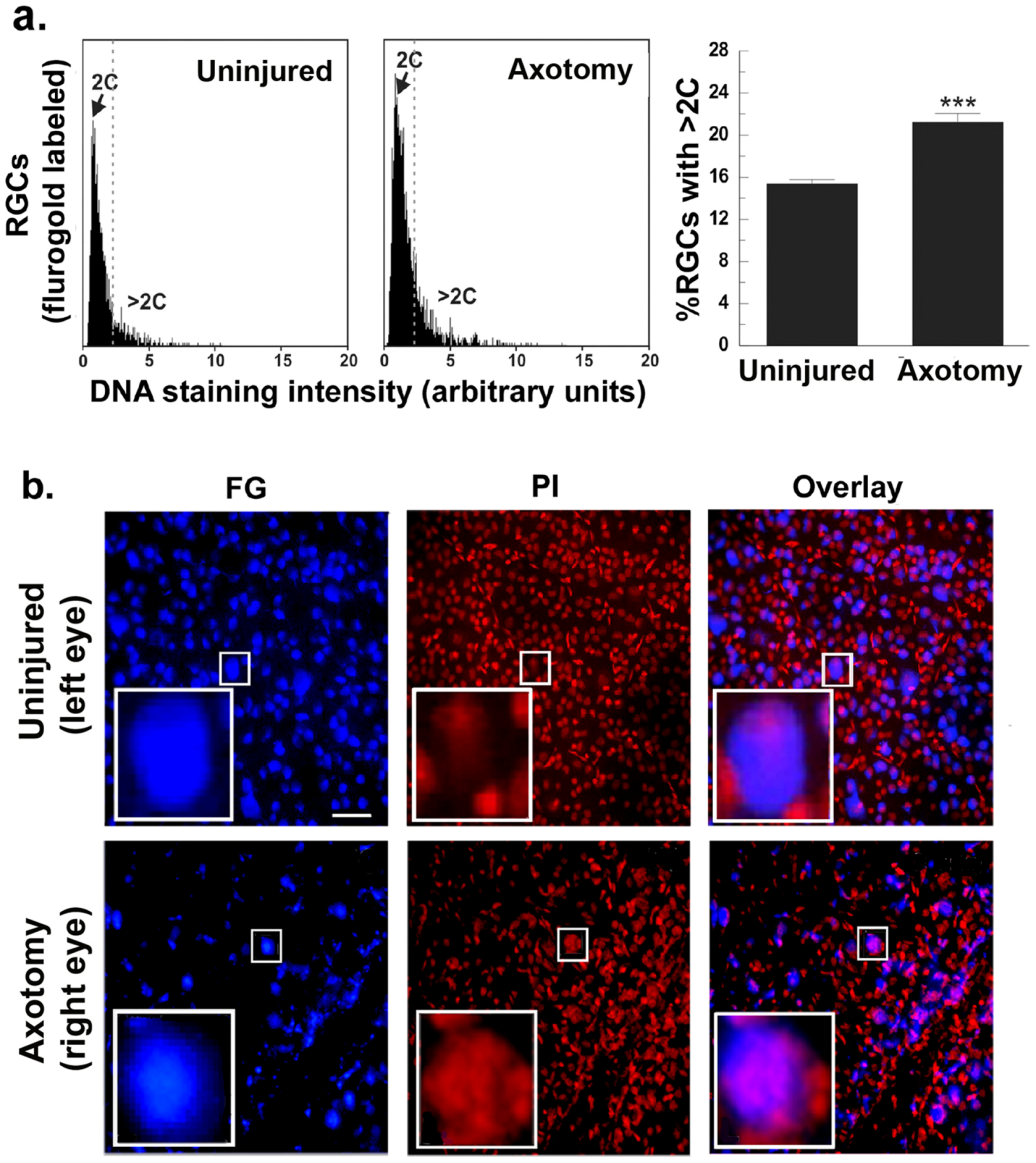
After axotomy some RGCs increase their DNA content. (**a**) RGCs were labeled with FG, and the retinas were flat-mounted on cover-slips and the DNA was stained with PI. Uninjured control and day 5 post-axotomy retinas were analyzed by SBC to quantify DNA intensity in FG-labeled RGCs. Axotomy significantly increases the percent of hyperploid RGCs, *** p<0.01. (**b**) Representative photomicrographs of flat-mounted retinas with images of FG and PI. The surrounding PI-labeled cells that are FG-negative, likely glia, are not RGCs by morphology and the fact that they are not labeled with FG. This PI-labeled population serves as background control. In each picture, the big white rectangle is an enlarged photomicrograph of the small white rectangle. Scale bar, 60 µm. FG: fluorogold; PI: propidium iodide.

We studied the DNA content of RGCs to determine whether they successfully progressed through the S-phase of the cell cycle. The DNA content of FG-labeled RGCs was analyzed quantitatively in retinas from uninjured control and at day 5 post-axotomy. The quantitative results using the SBC technique (see Methods) show that there is a significant increase in the subpopulation of hyperploid RGCs (>2C DNA content) (Figure 7a). Similar data were obtained on retinas prepared 3 days post-axotomy (data not shown).

The increase in DNA content of RGCs after axotomy was confirmed with a different technique, measuring PI intensity in FG-labeled retinas. In uninjured control retinas the PI intensity co-localized with FG (i.e. RGCs) was uniformly distributed. The PI intensity in RGCs was similar to other PI-labeled cells that are FG-negative and do not have RGC morphology. These neighboring cells are likely glia, and they serve as internal control.

In the uninjured retina ∼77% of the RGC were defined as small (<400 pixels) and 22% of the RGCs are defined as large (>400 pixels). The average PI intensity/pixel of these populations is not significantly different. In uninjured retinas, the average PI staining for small RGCs is 553±34 and for large RGCs is 438±67 (Table 2).

**Table 2:** Cell size distribution and propidium iodide (PI) intensity in the retina 5 days after axotomy.

		Small RGCs (100–400 pixels)	Large RGCs (400–1000 pixels)
**% RGCs per retina (±sem)**	Uninjured	77±1.9	22±0.25
	Axotomy	82±1.1	17±0.38
**Average PI intensity/pixel (±sem)**	Uninjured	553±34	438±67
	Axotomy	586±102	1135±85

Fluorogold^+^ PI^+^ RGCs were classified as small and large depending on their diameter in pixels. Cells with values between 100 and 400 pixels were arbitrarily considered “small” and cells with values between 400 and 1000 were consider “large”. More than 7000 cells per group were counted as described in the Methods and all retinal areas were averaged for each group.

At day 5 post-injury ∼82% of RGCs are small and ∼17% of RGCs are large, suggesting a relative reduction in the large RGC population. Concurrent to this, the average PI intensity/pixel was significantly elevated in large size RGCs (1135±85 in large RGCs *versus* 586±102 in small RGCs, *p*<0.01) (Table 2). Therefore, the average increase in DNA labeling was detected in RGCs that were of a relatively large-size (Figure 7b, Table 2). Similar data were obtained on retinas prepared 3 days post-axotomy (data not shown).

Together, these results provide a functional endpoint to cell cycle re-entry by RGCs, where 3–5 days post-axotomy large-size RGCs have more than 2C but less than 4C DNA content. These mature RGCs entered S-phase but only partially replicated DNA, and thus did not complete the S-phase nor did they enter mitosis.

### Inhibition of MAPK/ERK activity after injury reduces RGC death

**Figure 8:**
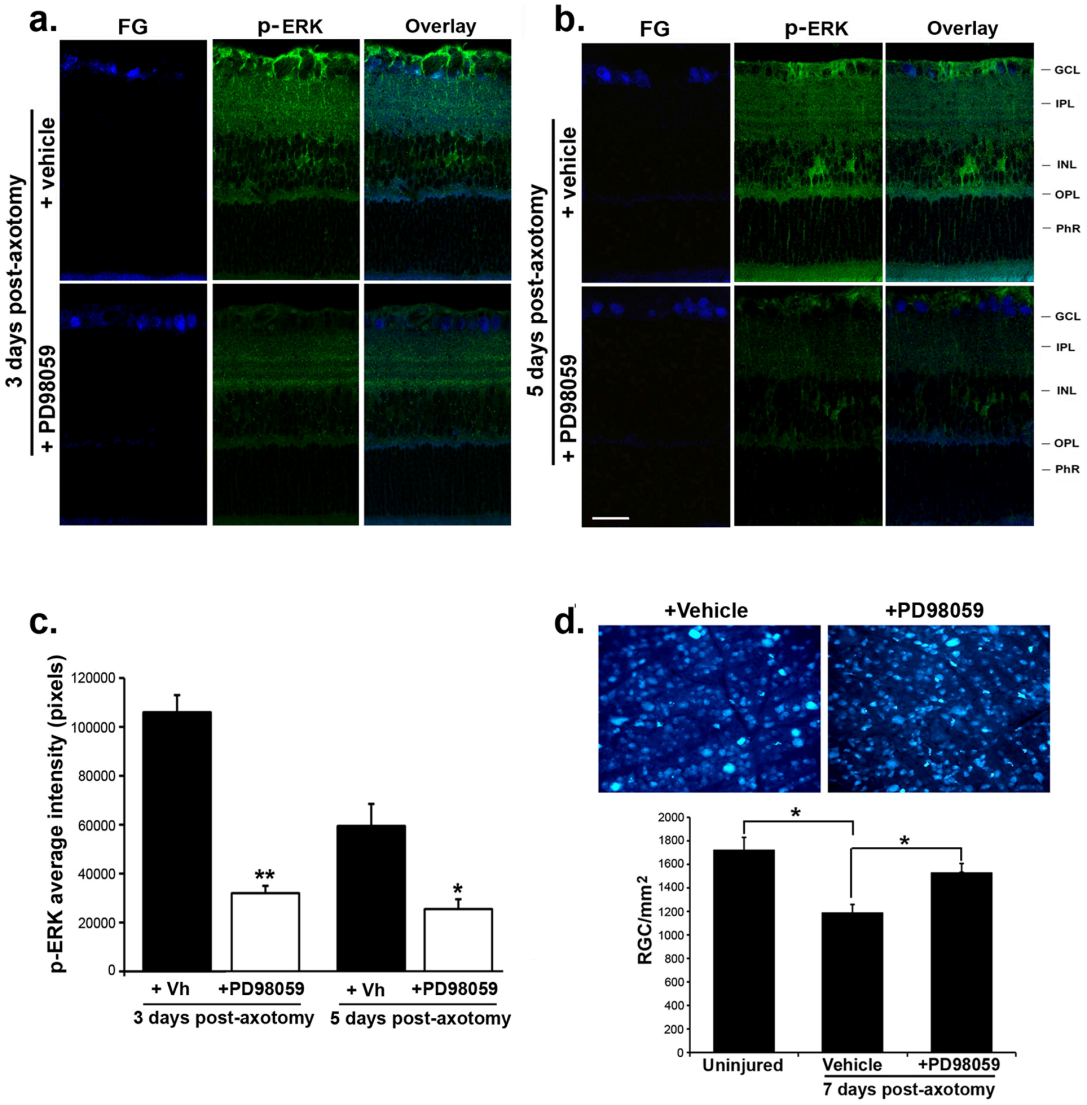
Treatment with PD98059 after axotomy inhibits p-ERK in glial cells and delays RGC death. p-ERK immunoreactivity in axotomized retinas from eyes treated with vehicle or PD98059 (**a**) retinas collected 3 days post-axotomy and (**b**) retinas collected 5 days post-axotomy. RGCs were labeled with FG prior to axotomy. In vehicle-injected eyes, p-ERK1/2 was increased in glial cells. In PD98059-injected eyes, p-ERK staining was reduced in the somata and in processes of Müller cell throughout all retinal layers. Scale bar, 30 µm. GCL: ganglion cell layer; IPL: inner plexiform layer; INL: inner nuclear layer; OPL: outer plexiform layer; PhR: photoreceptor layer. (**c**) Quantification of p-ERK intensity in vehicle or PD98059 treated eyes 3 days and 5 days post-axotomy (n = 4), * p<0.01 and ** p<0.001. (**d**) Representative pictures of FG-labeled RGCs in axotomized retinas treated with PD98059 or vehicle. Retinas were flat-mounted at day 7 post-axotomy and RGCs were quantified as described. The histogram is the quantification of FG-labeled RGCs in uninjured retinas and in axotomized retinas treated with PD98059 or vehicle, 7 days post-axotomy. Total of 48 images taken from n = 4 flat-mounted retinas per group. Values are number of RGC per mm^2^, * p<0.01.

Given that early after axonal injury there is a robust increase of p-ERK1/2 in glia and microglia cells but not in neurons (Figure 4), we tested the effect of a specific MAPK/ERK inhibitor on RGC death. PD98059 injected intravitreally at the time of ON axotomy prevented the p-ERK increase in glial/microglial cells and Müller cells. At post-axotomy days 3 and day 5, PD98059 abolished p-ERK staining in the somata and processes of glia/microglial cells in the inner and outer retinal layers (Figure 8a *versus* 8b and Figure 8c).

Inhibition of the MAPK/ERK pathway correlated within a significant decrease in RGC death. While at day 7 post-axotomy there is 39±8.7% RGC death, in the axotomized-PD98059-injected eyes there was only 15±1.73% RGC death (Figure 8d). These data suggest an RGC-death-promoting role for the rapid activation of the p-ERK cascade in glia after distal RGC injury.

## Discussion

Our data illustrates key molecular events that lead to RGC death after extra-retinal injury to neuronal fibers. First, injury rapidly activates retinal glia/microglia (p-Akt, p-S6, p-ERK1/2). Subsequently, the RGCs (but not glia) re-enter the cell cycle (Rb phosphorylation), reach the S-phase checkpoint (cyclin A activation), and enter the S-phase thereafter augmenting their DNA content (hyperploidy). Then RGCs die and their soma are eliminated; but this can be delayed with pharmacological inhibitors of the p-ERK cascade. This is the first evidence derived from a distal injury model that indicates RGCs increase their DNA content by undergoing cell cycle re-entry before their demise, and that p-ERK in glia may be a driver of the event.

### RGC injury activates glia and Müller cells

The PI3K/Akt and the Ras/ERK kinase cascades have been studied in other models of CNS trauma. However, this report is the first evidence of activation of p-Akt/p-S6 and ERK1/2 signaling pathways after distal ON injury.

We found a robust increase in p-Akt in Müller cells one day after RGC injury. Interestingly, p-Akt was compartmentalized towards the damaged RGC bodies; but p-Akt was not detected in the outer processes of Müller cells oriented towards photoreceptors. Glial responses vectorially oriented to the stressed RGCs may reflect a signal specifically arising from/to the injured neurons that is sensed by Müller cells. However, this is not a generalized mechanism because p-ERK-labeled glial processes were detected in fibers oriented towards injured RGCs but also towards the photoreceptor layer. We interpret this topographical distribution in glia as mechanism to integrate signals throughout the retina towards a biological endpoint, and this intriguing observation is currently under further study.

The majority of retinal diseases are associated with reactive gliosis [3], [22], [23]. The increase in glial p-Akt p-ERK and p-S6 was accompanied by signs of reactive gliosis, hypertrophy and swelling of cellular processes 3 days post-axotomy. However, we did not detect p-Rb, or cyclin A in Müller cells. Thus, while glial cells became activated and hypertrophic, they do not appear to proliferate in this time frame. Glial cell proliferation may occur at later stages or by migration of microglia or astrocytes.

In healthy states glia play a supportive role for RGC maintenance, survival, and function. Upon neuronal injury, even distant neuronal injury, that role switches to one where glia are induced to destroy and remove the RGCs. Indeed, delayed RGC death was achieved by pharmacological inhibition of p-ERK [9], [24]. Here we further demonstrate that PD98059 is inhibiting p-ERK activated in glia and Müller cells early after RGC injury, rather than p-ERK in RGCs (which is undetectable in injured RGCs), indicating a glia-to-neuron paracrine effect.

We and others have reported that glia can be stimulated to release a gradient of soluble factors to kill retinal neurons: proNGF [5], [25], TNF-α [7], [26], reactive oxygen species and other toxic molecules [3], [27]. Likewise, we speculate that soluble or membrane-bound factors or signals from RGCs may inform glia of an injury, but these signals remain elusive.

### RGC injury activates cell cycle in post-mitotic neurons

p-S6 is detected in the RGCs of uninjured control retinas, but labeling completely disappeared in RGCs three days after axotomy. Reduced p-S6 in RGCs would impact on the mTOR-dependent pathways [14], and on regulation of global translation of mRNAs [15]. Presumably, this could serve to selectively translate mRNAs or to dedicate resources for cellular maintenance/survival after RGC injury [28].

In axotomized eyes, RGCs showed a robust increase in cytoplasmic p-Rb, suggesting that RGCs re-entered the cell cycle. Cell cycle proteins are normally down-regulated in post-mitotic neurons [11]. However, given that Rb and E2F1 also regulate the expression of proteins involved in apoptosis and autophagy [29], cell cycle re-activation in post-mitotic neurons can ultimately cause apoptosis instead of proliferation [30], [31].

The level of E2F1 immunoreactivity was similar in RGCs from both healthy and injured eyes. It is likely that the antibody recognizes E2F1 when it is still part of the E2F1/Rb complex. Nevertheless, the significance is that after axotomy phosphorylation of Rb can result in the dissociation of the Rb/E2F complex and activation of E2F1 transcription factor, which could subsequently lead to cell cycle re-entry, progression through G1-to-S phase, and eventual RGC death.

After axotomy we did not observe changes in the expression of cyclin D3. Although cyclin D3 can form active kinase complex with the cyclin-dependent-kinase (CDK) 4 or CDK 6, resulting in the phosphorylation of Rb protein [32], one study demonstrated that increased CDK4 is sufficient to phosphorylate Rb and drive neurons into apoptosis [33]. Other members of the cyclin D family may participate in this process. Indeed, cyclin D1 has been shown to be upregulated in apoptotic retinal neurons after ischemia reperfusion [34]–[36]. Hence, it seems that the activation or expression of cell cycle proteins does not necessarily cause canonical cell cycle re-entry. Rather, the expression and activity are aberrant and occurring out of sequence [37].

### Cell cycle and neuronal death in injured RGCs

Studies have shown correlations between dysregulation of cell cycle-regulatory proteins and neuronal death after acute trauma or stroke damage, and also during chronic neurodegenerative diseases [2], [38]. These studies indicate that an aberrant cell cycle re-entry might be a common pathway leading to neuronal death. In our work we further showed that injured RGCs not only re-enter the cell cycle but progress through it and reach the S-phase, as demonstrated by activation of cyclin A. Then, injured RGCs increase their DNA content (DNA hyperploidy, >2C).

DNA hyperploidy in RGCs has been reported during embryonic development. However there is an important distinction with respect to the adult RGCs. In the RGCs of the adult retina Rb-phosphorylation precedes hyperploidization. In clear contrast, during RGC embryonic development, the tetraploid embryonic RGCs are Rb-negative [12]. Hence, there may be a different mechanism leading to DNA hyperploidy after pathological injury of mature neurons *versus* the process of maturation and selection of developing retinal neurons. In part, this may be related to the fact that mature differentiated RGCs are already selected, they are functional, and they have made synaptic connections to the brain cortex.

Endoreduplication [39], [40], characterized by DNA synthesis in absence of cell division, has been demonstrated to yield tetraploid embryonic RGCs [12]. Among other mechanisms that could generate hyperploid mature RGCs, entosis [41], and cell fusion [42] are noteworthy. However, endoreduplication leading to >2C hyperploidy is more likely given that RGCs undergo cell cycle re-activation.

Cell cycle re-entry and DNA hyperploidy 3–5 days post-axotomy is primarily detected in large-sized RGCs. Related to this observation, reports have shown that large RGCs are more vulnerable to death in models of axotomy and in glaucoma [43]–[45], and mature neurons in the cell cycle are destined to die [46]. However, we do not know whether signals promoted by axotomy cause cell cycle re-entry in a pre-existing subpopulation of large diploid RGCs, or whether a subpopulation of small-sized RGCs undergo hypertrophy before cell cycle re-entry. Either or both options are possible, because differential gene expression between large and small RGCs [47] could make each sub-population differentially susceptible to stress.

### Conclusions and hypothetical model

Extra-retinal injury to RGCs initiates signaling cascades. Glia are activated by signaling molecules or by membrane bound effectors from RGCs. Glia then trigger the Akt/ERK and p-S6 signaling pathways, in a fairly compartmentalized fashion directed towards RGCs. Ultimately, RGCs enter into an aberrant cell cycle through p-Rb/E2F/cyclin A, increase their cell body mass, increase their DNA content, and undergo cell death. Interfering with the MAPK/ERK cascade by using PD98059, reduces p-ERK in glial cells, and protects RGCs against cell death. Others have shown that cell cycle inhibitors protect against neuronal death due to brain trauma [46], and against neuronal death in the retina after ischemia/reperfusion [34].

**Figure 9:**
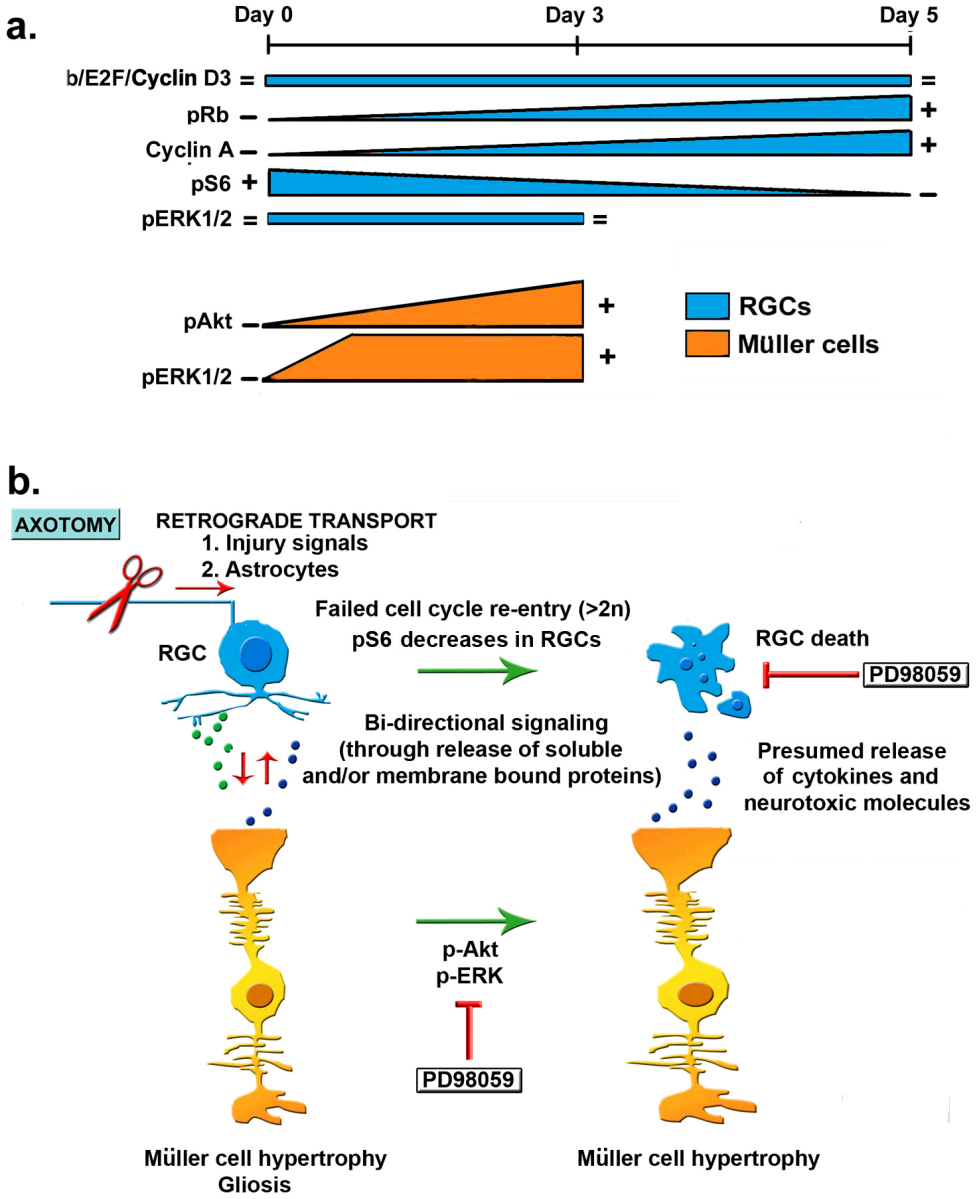
Hypothetical model of events leading to RGC death after axotomy. (**a**) Time-dependent retinal changes for cell cycle markers in RGCs (blue) and Müller cells (orange) after optic nerve transaction (extra-retinal damage). (**b**) Neuro-glia interactions. Optic nerve transaction induces retrograde transport of “injury signals” and migration of reactive astrocytes to the retina. Injured RGCs and glial cells are in intimate contact and also communicate by releasing soluble molecules. The interaction may be bi-directional. Early after RGC injury, glial cells rapidly activate p-Akt, and p-ERK pathways. The p-Akt response in Müller cells is specifically oriented towards the injured RGCs. Early after RGC injury glia do not undergo Rb phosphorylation or cell cycle re-entry, but they do undergo hypertrophy. Shortly thereafter, and before significant RGC death, the injured RGCs decrease p-S6, phosphorylate-Rb, re-enter the cell cycle and undergo DNA synthesis. Later the RGCs undergo frank cell death and their somata are cleared from the retina, but RGC death can be delayed by inhibiting p-ERK in glial cells.

We propose a hypothetical model to integrate these observations (Figure 9). Delayed kinetics of RGC death after axotomy have distance from cell body as a variable, with the “injury” signals traveling retrogradely along the proximal fibers towards the RGC soma. Also reactive astrocytes can travel along the optic nerve towards the retina. However, the resident glia (e.g. Müller cells) do not appear to undergo proliferation in the time frame, based on the absence of markers of cell-cycle re-entry. These cells do undergo hypertrophy, which is consistent with the activity of p-S6, p-Akt and p-Erk1/2.

It is also possible that the signals to activate glia are relayed by the RGCs themselves (e.g. through membrane-bound factors or ions or a soluble factor gradient) and/or by new astrocytes that migrate into the injured retina. The concept of a gradient is attractive because the p-Akt distribution within Müller cells was polarized towards the RGCs, whereas p-Erk was not polarized. Not surprisingly, the Müller cell hypertrophy is also “unidirectional” (e.g. it occurs towards the RGCs and not towards the photoreceptors).

We and others have shown that after axotomy activated glia produce neurotoxic factors such as TNF-α and pro-NGF, and these factors kill RGCs [7], [25]. However, glia can also play an essential supportive role for RGCs. We speculate that the balance of the quality and/or the intensity of the functional interactions between injured neurons and glia may be the arbiter of whether glia will protect or kill the damaged neurons.

In this context, the neurotrophin system represents an elegant putative mechanism because in the retina the precursor pro-neurotrophins kill RGCs while the mature neurotrophins play a role in maintenance [7], [25]. Further studies will address this hypothesis.
